# Pore-scale experimental investigation of oil recovery enhancement in oil-wet carbonates using carbonaceous nanofluids

**DOI:** 10.1038/s41598-020-74450-w

**Published:** 2020-10-16

**Authors:** Bingjun Zhang, Abdelhalim I. A. Mohamed, Lamia Goual, Mohammad Piri

**Affiliations:** Department of Petroleum Engineering, Center of Innovation for Flow Through Porous Media, University of Wyoming, Laramie, WY 82071 USA

**Keywords:** Environmental sciences, Hydrology, Energy science and technology, Engineering, Materials science, Nanoscience and technology

## Abstract

This study investigates the pore-scale displacement mechanisms of crude oil in aged carbonate rocks using novel engineered carbon nanosheets (E-CNS) derived from sub-bituminous coal. The nanosheets, synthesized by a simple top-down technique, were stable in brine without any additional chemicals. Owing to their amphiphilic nature and nano-size, they exhibited dual properties of surfactants and nanoparticles and reduced the oil/brine interfacial tension (IFT) from 14.6 to 5.5 mN/m. X-ray micro-computed tomography coupled with miniature core-flooding was used to evaluate their ability to enhance oil recovery. Pore-scale displacement mechanisms were investigated using in-situ contact angle measurements, oil ganglia distribution analysis, and three-dimensional visualization of fluid occupancy maps in pores of different sizes. Analysis of these maps at the end of various flooding stages revealed that the nanofluid invaded into medium and small pores that were inaccessible to base brine. IFT reduction was identified as the main displacement mechanism responsible for oil recovery during 1 to 8 pore volumes (PVs) of nanofluid injection. Subsequently, wettability alteration was the dominant mechanism during the injection of 8 and 32 PVs, decreasing the average contact angle from 134° (oil wet) to 85° (neutral wet). In-situ saturation data reveals that flooding with only 0.1 wt% of E-CNS in brine resulted in incremental oil production of 20%, highlighting the significant potential of this nanofluid as a recovery agent.

## Introduction

Chemical flooding processes usually entail the injection of additives along with brine to enhance the productivity of oil-bearing subsurface formations^1,2^. Surfactants are often added to reduce the interfacial tension (IFT) between oil and brine^3–5^, which would significantly decrease the threshold capillary forces in porous media. The IFT reduction as well as the emulsification effect of surfactant solutions contribute to a better mobilization of residual oil through pores and throats, yielding a higher recovery^6–8^. Nanoparticles (NPs), on the other hand, are commonly injected because of their small size and large surface area to volume ratio, which endows them with high interfacial activity^9–13^. Several studies have investigated the injection of NPs with brine, polymer, and even foams to enhance oil recovery^14,15^. While they often exhibit an insignificant impact on IFT, NPs can alter the wettability of mineral surfaces and hence decrease the capillary forces responsible for trapping oil inside the pores^16,17^. The mechanism of wettability alteration may be triggered by nanoparticle adsorption on the rock or their ability to displace oil from mineral surfaces due to the structural disjoining pressure^18–21^.

Despite their advantages, chemical flooding processes are limited by the high cost of additives and their potential losses through adsorption^22–24^. Low retention by the rock is also preferred to reduce pore plugging^25,26^. Traditional NPs, which consist mostly of metal oxide dispersions, may become colloidally unstable under reservoir conditions and cause formation damage^16,27^. Their field application may be hampered by water contamination or catalyst poisoning in downstream operations^28,29^. Therefore, the need for additives with dual properties of surfactant and NPs for enhanced oil recovery (EOR) has prompted the development of functionalized nanomaterials with amphiphilic nature and a superior ability to withstand harsh reservoir conditions. The design and improvement of these high-performance functional fluids rely on the preparation and organization of distinct chemical structures with key features engineered on nanometer to micrometer length scales. As a result, their properties are highly sensitive to strong intermolecular interactions among dissolved or dispersed constituents. The ability to synthesize complex formulations and understand their structure–function relationships has several merits as it allows to customize injected fluids to specific conditions for optimum stability and efficiency, and provides a sound platform for the design of innovative and environmentally friendly products.

Among functionalized nanomaterials, silica- and graphene-based nanosheets were proposed as a new generation of EOR additives. Both types of nanosheets were found to lower IFT and alter wettability, leading to a considerable increase in oil recovery^30–35^. Nanosheets form climbing films at the oil–water interfacial area, which encapsulate and detach oil from the rock surfaces. The elastic film at the interface could resist bending and self-assemble after rupture^33,35^. Furthermore, oxygen-containing groups in functionalized silica-graphene nanohybrids contributed to IFT reduction and increased macroscopic sweep efficiency. It was postulated that the electrostatic interactions between these groups and positively charged sodium in brine improved the stability of oil-in-water emulsions^17^. While insightful, most of these mechanisms were inferred empirically from macro-scale experiments. Moreover, there was no direct experimental evidence of rock-fluid interactions at the pore scale, which greatly limited the interpretation of their effects on EOR.

The understanding of pore-scale displacement mechanisms that govern fluid flow and transport in the presence of amphiphilic nanosheets could be significantly improved by conducting micro-scale experiments under high-pressure and high-temperature (HPHT) conditions. These flow tests, combined with nondestructive x-ray micro-computed tomography (micro-CT), enable the study of fluid–fluid and fluid-rock interactions by visualizing pore space topology and structure as well as fluid occupancy at the micron scale. Yet, to the best of our knowledge, they have never been used in the past to study carbonaceous nanoparticles under HPHT conditions. Computation of saturation profiles and in-situ wettability distributions could be further utilized to inform the development of novel nanomaterials with enhanced EOR performance^36–38^.

In this work, we use a sub-bituminous coal from Wyoming to synthesize novel functionalized graphene-based nanosheets. Graphene oxide (GO) is first extracted using a modified Hummer’s method then partially grafted with an alkylamine using starch as a template^34^. The reaction products are engineered carbon nanosheets (E-CNS) with an amphiphilic character that endows them with both surfactant-like and nanoparticle-like properties^39^. The impact of E-CNS on the pore-scale displacement mechanisms of crude oil in a natural carbonate rock is probed under elevated pressure and temperature conditions using a state-of-the-art micro-CT apparatus integrated with a miniature core-flooding system^40,41^. By analyzing the pore-scale fluid occupancy maps and in-situ wettability before and after nanofluid flooding, new insights are gained on the superior ability of the carbonaceous nanofluid to alter the wettability of the rock and displace oil from pores of various sizes.

## Materials and methods

### Materials

Powder River Basin (PRB) coal from Wyoming was sieved through a mesh size of 300 and used for the synthesis of graphene oxide. Potassium permanganate (KMnO_4_) and sodium chloride (NaCl) were obtained from Fisher Scientific in crystalline form. Sulfuric acid (95–98%), hydrogen peroxide (30 wt% in H_2_O), dichloromethane, toluene, ethanol, sodium nitrate, dodecylamine (DDA, > 99.5%), and Alconox were all procured from Sigma Aldrich and used as received. All the dispersions were prepared using a CL-334 horn of Q500 Qsonica sonicator. For the core-flooding experiments, we used a light crude oil from the Permian Basin in Texas, USA. The oil was centrifuged at 6,000 rpm for one hour and then filtered through a 0.5 µm filter to remove any solid contaminants. About 6 vol% of diiodomethane (> 99%, Fisher Scientific) was added to the oil to enhance the contrast between the aqueous solution (i.e., the base brine and E-CNS) and the oil phase in the micro-tomographic images. The base brine was prepared by dissolving 30 mM NaCl in distilled water (DI) with a conductivity of 1.48 × 10^−4^ s/m. The brine concentration was selected as the highest brine concentration under which the E-CNS nanofluid dispersion could retain its stability for at least two weeks without adding any surfactant or stabilizer. This concentration was comparable to those frequently used in slick water hydraulic fracturing and other nanoparticle studies^42,43^.

### Rock

The natural rock sample used in this study is a carbonate outcrop obtained from Fond Du Lac (FDL) formation located in Wisconsin, USA. This rock was selected because it contained less microporosity than most carbonates^44,45^, which reduced errors in the estimation of porosity and in-situ fluid saturation by X-ray microtomography. Two cores with 1.5 inch in diameter and 6 inch in length were drilled from large blocks and then cut into four 1.5 inch in diameter, 3 inch in length core plugs. The parent core plugs were washed with tap water and placed in an oven at 110 °C for two days. The porosity and permeability of the core plugs were determined by a Coretest AP-608 automated porosimeter at a confining pressure of 500 psi. The basic petrophysical properties of the core plugs are listed in Table S1. Core sample No. 4 with the highest permeability and porosity was selected as the parent sample to drill the 9.8 mm in diameter, 68.4 mm in length miniature core sample for the flooding experiments. After loading the miniature sample to the core-flooding system, the absolute brine permeability of the core was measured to be 35 mD from Darcy’s law by calculating the slope of steady-state pressure drops measured across the core at different constant flow rates. Helios Nanolab Scanning Electron Microscope (SEM) from Thermo Fisher Scientific was used to image the rock, and its energy dispersive x-ray spectrometer (EDX) module was adopted to analyze the elemental composition. A micrograph of the rock is shown in Figure S1(a), while its elemental composition is listed in Table S2. FDL outcrop is a carbonate with a 1:3 ratio of CaCO_3_ to MgCO_3_, which is typically a dolomite. This mineralogy was further confirmed in Figure S1(b) by Quantitative Evaluation of Minerals by Scanning Electron Microscopy (QEMSCAN 650F, Thermo Fisher Scientific) analysis. The x-ray source was optimized at 25 kV and 6.2 nA, and a Species Identification Protocol “HMS Primary v 1.0" was used to convert raw data to 3 × 3 mm^2^ mineralogy maps with an optical resolution of 0.73 µm per pixel. The pore size distribution of the rock was also evaluated using a micro-CT scanner (HeliScan, Thermo Fisher Scientific) at a resolution of 2.366 µm. The x-ray source was operated at a tube voltage and current of 100 kV and 55 μA, respectively. The scanned images were reconstructed by Q-Mango platform and then analyzed using Avizo Fire 9.4 software.

### Synthesis and characterization of E-CNS

**Figure 1:**
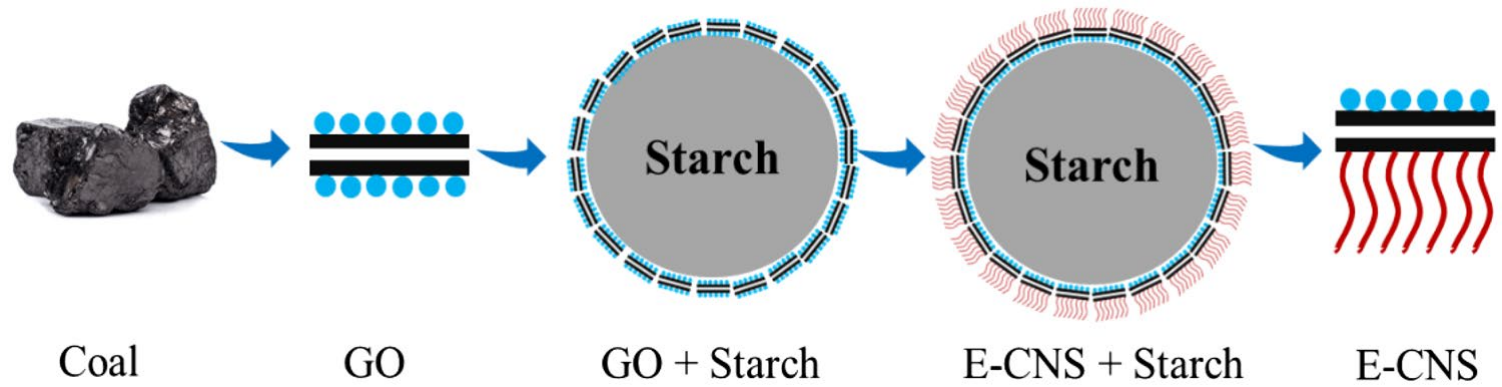
Schematic of E-CNS synthesis procedure. The blue circles represent the oxygen-rich functions of GO while the red lines represent the hydrophobic chains of DDA.

For the synthesis of E-CNS, 100 cm^3^ dispersion of coal-derived GO (1 mg/ml in brine) was added to a mixture of 20 g soluble starch in 250 cm^3^ DI water. This mixture was stirred for 12 h to allow the GO to adsorb on the starch microspheres by hydrogen bonding. The starch microspheres were then filtered with an 8-micron filter paper to remove the unabsorbed GO. It was further washed with 100 cm^3^ ethanol. The starch microspheres were re-dispersed in 250 cm^3^ ethanol. 300 mg of DDA in 50 cm^3^ ethanol solution was added to the starch, and the mixture was stirred for 20 h to allow the amine to react with the exposed side of GO. This mixture was filtered with an 8-micron filter paper and washed with an excess of ethanol to remove the unreacted DDA. The GO-DDA coated starch microspheres were then subjected to heating (60 °C) and sonication cycles to detach the GO-DDA from the starch microspheres. This fluid system separated into two phases overnight. The starch microspheres settled in the bottom while the E-CNS remained dispersed in ethanol in the top layer. The dispersion was separated and dried to extract the E-CNS nanoparticles. A schematic of the synthesis procedure is provided in Fig. 1. More details on the synthesis and characterization of these nanoparticles can be found elsewhere^39^. Micrographs of the E-CNS were obtained by Titan Environmental Transmission Electron Microscope (E-TEM, Thermo Fisher Scientific). Ted Pella copper TEM grids were first dipped into the nanofluid and then dried overnight in a vacuum oven. The zeta potential and particle size distribution of E-CNS dispersions were obtained using ZetaPALS Brookhaven analyzer with a 40 mW red diode laser and a nominal 640 nm wavelength. The dispersions were prepared by mixing 0.01% of E-CNS in brine for 30 min using a probe sonicator.

### Dynamic interfacial tension

The dynamic interfacial tension between E-CNS nanofluid-in-brine and Permian crude oil was measured with a pendant drop method. The setup includes a well-sealed Hastelloy cell with observation windows for camera imaging, a dual cylinder Quizix 5000 syringe pump (Chandler Engineering) for liquid delivering, a High-definition (HD) camera for image acquisition, a vibration-free work bench to support the cell, and other accessories. The fluid densities were measured by an Anton Paar PMA 5100 densitometer. More details on the apparatus can be found elsewhere^46^. Before any experiment, the system was first cleaned with toluene, methanol, and distilled water, then dried with nitrogen to prevent any possible contamination. The oil/brine IFT was measured in the Hastelloy cell. However, the IFT between E-CNS nanofluid and crude oil could not be measured in the cell because the backlight was unable to penetrate through the translucent E-CNS dispersion. To resolve this issue, a cuvette was fabricated to minimize the path length to allow the light to pass through the nanofluid to the camera. A small drop of oil was injected into the cuvette filled with nanofluid, and IFT was estimated from the asymmetric drop shape analysis. Each measurement was repeated three times to ensure reproducibility of the data.

### Core-flooding procedure

Information on the core-flooding system, data acquisition, and image analysis procedures are provided in the Supplementary Information. A detailed schematic diagram of the experimental setup is shown in Figure S2(a)^41,47,48^. The core-flooding procedure consists of six different steps (Figure S2(b)). (i) The miniature core sample was first placed in the vertically-oriented core holder, then a confining pressure of 200 psi was applied, thereby compressing the rubber sleeve surrounding the core to force the fluid to flow through the core without bypassing. Thereafter, CO_2_ gas was injected into the core for 30 min at a pressure of 40 psi to remove air inside the core. (ii) A vacuum pump was connected to the core holder to remove the CO_2_ gas inside the sample. The vacuum was then continued for 12 h to remove previously injected CO_2_. At this stage, the core was scanned to collect dry reference images that were used to map the pore space and generate porosity distribution along the field of view (FOV). Brine was then injected and the pore and confining pressures were simulateniously increased up to 300 and 500 psi, respectively, to maintain a 200-psi net confining pressure. Subsequently, the temperature of the system was gradually increased to 50 °C (122 °F). More than 100 PVs of brine were injected at a flow rate of 0.05 cm^3^/min to dissolve and flush any trapped CO_2_. The absolute permeability of the rock to brine was measured by recording the differential pressure across the core at different flow rates. The back-pressure pump was kept at a constant pressure receiving mode to maintain the pressure near the outlet of the sample at 300 psi. (iii) Primary drainage was commenced by injecting the doped crude oil at a flow rate of 0.05 cm^3^/min. The oil injection was continued until the flow reached steady state. This condition was determined by recording a stable differential pressure across the core and obtaining similar saturations from two consecutive scans. A scan was then taken to record the initial water saturation (S_wi_). (iv) A dynamic aging process was initiated by injecting the oil at a low flow rate of 0.002 cm^3^/min. A particularly low flow rate was used to prevent any significant changes in the established S_wi_. The aging process was continued for three and a half weeks to alter the wettability of the dolomite from initially water-wet to oil-wet. The pressure drop across the core was monitored and scans were taken at the end of the aging process. The aging continued until the in-situ contact angles measured in the last two scans were comparable. Then the scan was used as a reference for subsequent tests. (v) Waterflooding was performed by injecting base brine at a flow rate of 0.005 cm^3^/min to establish a capillary-dominated flow regime (with a capillary number of 5.3 × 10^−8^). The core was scanned consecutively after 1, 8, and 32 PVs of brine injection and in-situ contact angles were measured at each of these flooding stages. (vi) A secondary oil injection was conducted after the waterflooding processes. As the waterflooding had no impact on contact angle, the goal of this step was to restore the oil saturation to the value obtained in Step (iv). This step is significant as it ensures a similar initial condition for the subsequent E-CNS nanofluid flooding. The oil was injected at a flow rate of 0.05 cm^3^/min for 72 h. A scan was conducted to ensure that the initial oil saturation and wetting condition were comparable to those of Step (iv). (vii) After restoring the core to conditions similar to those before waterflooding, the nanofluid flooding was performed using 0.1 wt% of E-CNS in brine. The experiment was conducted at the same conditions as those in Step (v). The same flow rate of 0.005 cm^3^/min was also selected to ensure a capillary-dominated flow regime. The core was scanned after the injection of 1, 8 and 32 PVs. The in-situ contact angles were measured at the end of each of these E-CNS flooding steps. Bump tests were carried out after both base brine and nanofluid floodings at a flow rate of 0.006 cm^3^/min to confirm that we had reached the residual oil saturations and no more oil could be produced.

## Results and discussion

### Synthesis and characterization of E-CNS

**Figure 2:**
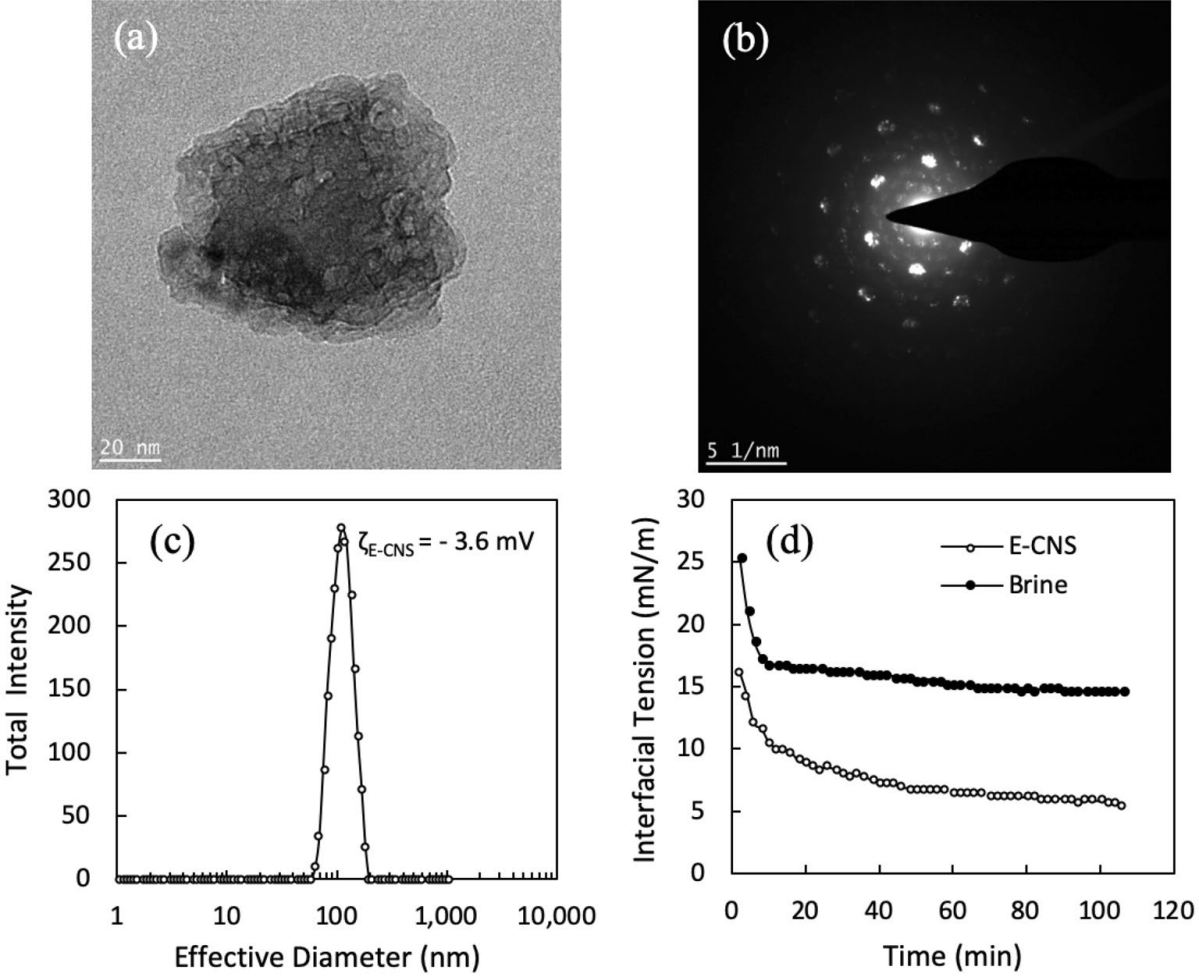
(**a**) TEM micrograph of E-CNS, (**b**) diffraction pattern of E-CNS, (**c**) particle size distribution by DLS and (**d**) dynamic IFT between the crude oil and base brine with and without E-CNS^39^.

Engineered carbon nanosheets were synthesized in two consecutive steps. First, graphene oxide nanosheets were extracted from coal by a modified Hummer’s method, then asymmetrically modified using soluble starch as a template^34,39^. One side of the nanosheets was protected by the template while the other side reacted with dodecylamine, leading to an amphiphilic structure. The TEM micrograph in Fig. 2a shows the two-dimensional sheet-like and layered structure of E-CNS, where the basal planes slip out of alignment in a slightly staggered angle. We estimated the number of layers per nanosheet to be in the range of 5–10 based on the diffraction pattern in Fig. 2b^39^. Dynamic light scattering (DLS) of E-CNS dispersions in brine revealed an average diameter of 110 nm and a zeta potential of − 3.6 mV (Fig. 2c). Considering a thickness of less than 10 nm, E-CNS are typically two-dimensional nanomaterials. With their flat shape and asymmetrical hydrophobicity on different sides, E-CNS can better position themselves at the oil/water interface compared to spherical metal oxide nanoparticles. As a result, they can lower the IFT between brine and crude oil by 62% (i.e., from 14.6 to 5.5 mN/m) at ambient conditions, as seen in Fig. 2d. This is an idiosyncratic property of E-CNS since most nanoparticles show little effect on IFT^42^.

**Figure 3:**
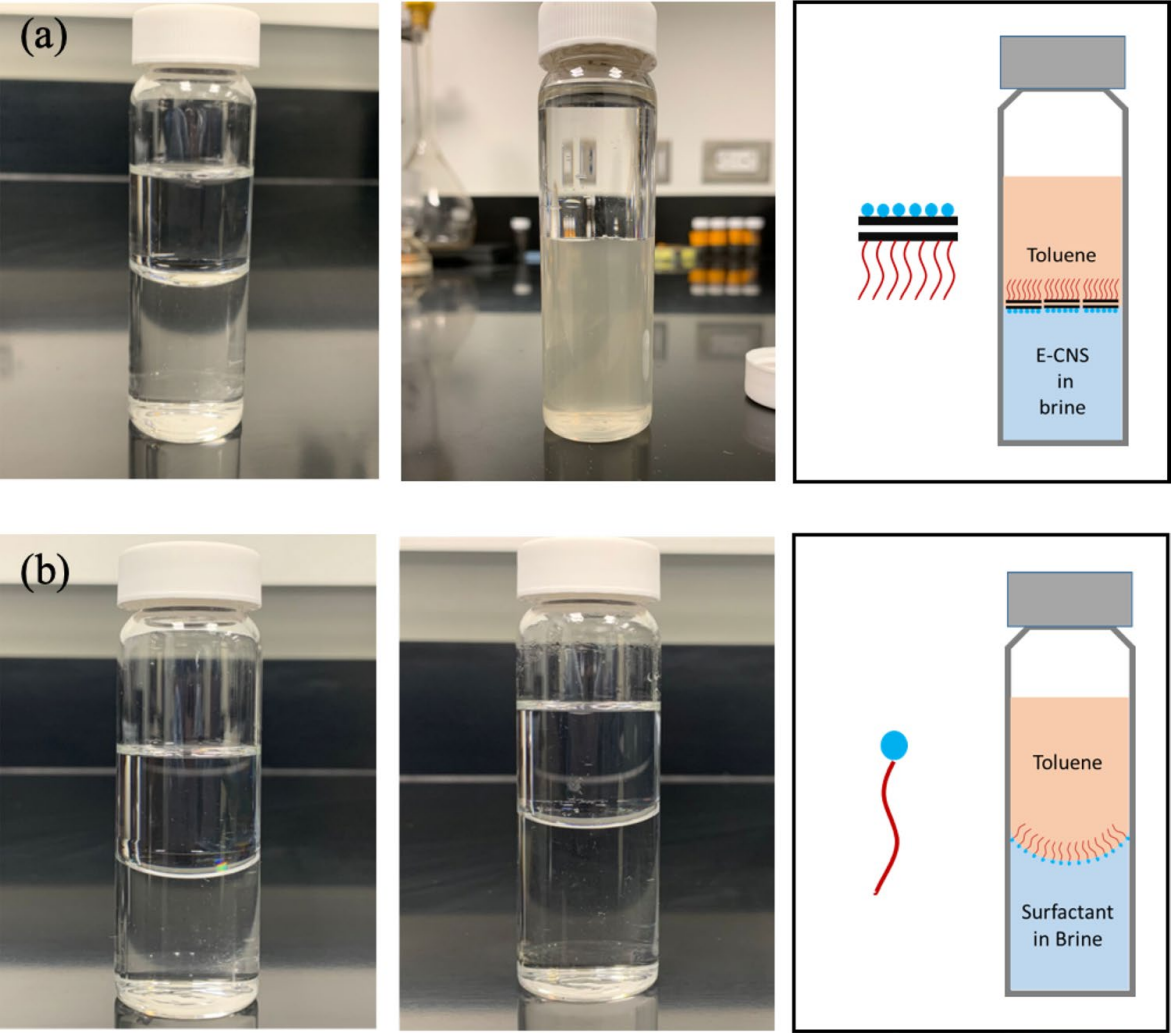
Bottle tests showing the meniscus between toluene and water before (left) and after (right) the addition of (**a**) E-CNS and (**b**) Alconox surfactant to brine. The blue circle represents the hydrophilic head while the red line represents the hydrophobic tail of the chemical additive.

The amphiphilic nature of the nanofluid is further confirmed through slim-bottle tests where toluene (organic phase) and distilled water (aqueous phase) are first added to two glass vials. The curvature in the three-phase contact regions of both vials shows a concave meniscus prior to introducing any chemical agents. After the addition of E-CNS to the first vial, the meniscus gradually flattens due to the preferential orientation of E-CNS at the interface (Fig. 3a). The nanosheets tend to have their hydrophobic side facing upwards towards the toluene, while their hydrophilic side is facing downwards towards the water. This behavior is not observed in the second vial where Alconox surfactant had been added (Fig. 3b). Although surfactants orient themselves similarly at interfaces, they do not flatten the curvature due to the absence of a graphitic backbone in their structure.

Stability tests with solutions of 0.1 wt% of E-CNS in brines of different salinities were previously performed and are presented elsewhere^39^. We found that the nanofluid could maintain its colloid stability at 1,500 ppm brine salinity and 50 °C for at least 15 days. This stability was mainly controlled by the interplay between attractive forces (i.e., van der Waals and hydrophobic) and repulsive forces (i.e., electrostatic and steric). This balance may shift in high salinity brines due to double layer compression. In this case, surfactants are needed to ensure the dispersion of E-CNS while maintaining their unique interfacial behavior.

### Rock characterization

**Figure 4:**
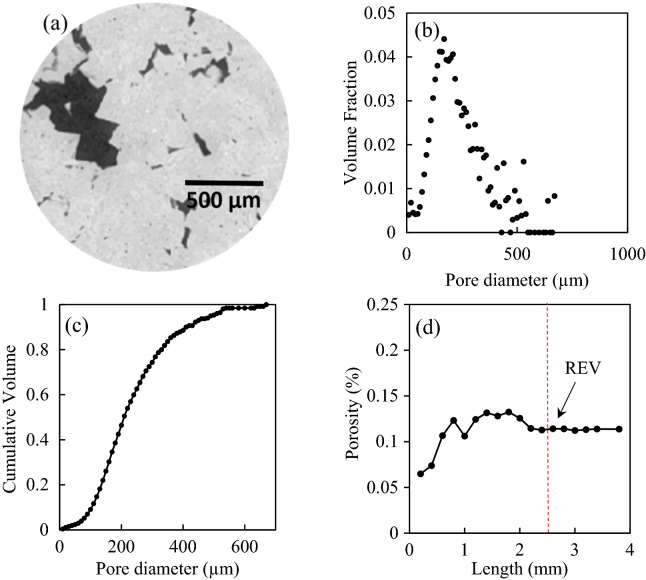
Pore-space properties of FDL limestone; (**a**) 3D X-ray microtomographic image. Variations in (**b**) volume fraction and (**c**) cumulative volume with changes in pore diameter; and (**d**) porosity-based REV.

The pore topology and size distribution of FDL carbonate were obtained from micro-tomography images and are displayed in Fig. 4a,b. The rock sample has a wide pore size distribution with a peak at approximately 200 μm. Based on the cumulative volume profile shown in Fig. 4c, the pores could be divided into three categories with comparable volumes. Small pores are recognized as those with a diameter smaller than 170 μm, which take 34.62% of the total pore volume. Similarly, medium pores are those with a diameter between 170 and 270 μm, which represents 33.53% of the total pore volume. Large pores are those with a diameter larger than 270 μm, taking 31.85% of the total pore volume. To determine whether the scanned field of view (FOV) was within porosity-based representative elementary volume (REV), the variation of porosity with changes in volume was investigated after the injection of several pore volumes of brine. The porosities of cubical sub-volumes of the FOV with various lengths from 0.2 to 3.8 mm were calculated. The sub-volume beyond which the petrophysical properties showed no significant fluctuation was considered as the REV. Figure 4d shows that the porosity varied significantly until the cubical sub-volume side length exceeded 2.4 mm, suggesting a REV value of 2.4 mm. This was significantly smaller than the size of the FOV (7.0 mm in diameter and 5.2 mm in length). Thus, the scanned volume was representative of the overall rock sample.

### Core-flooding tests

**Figure 5:**
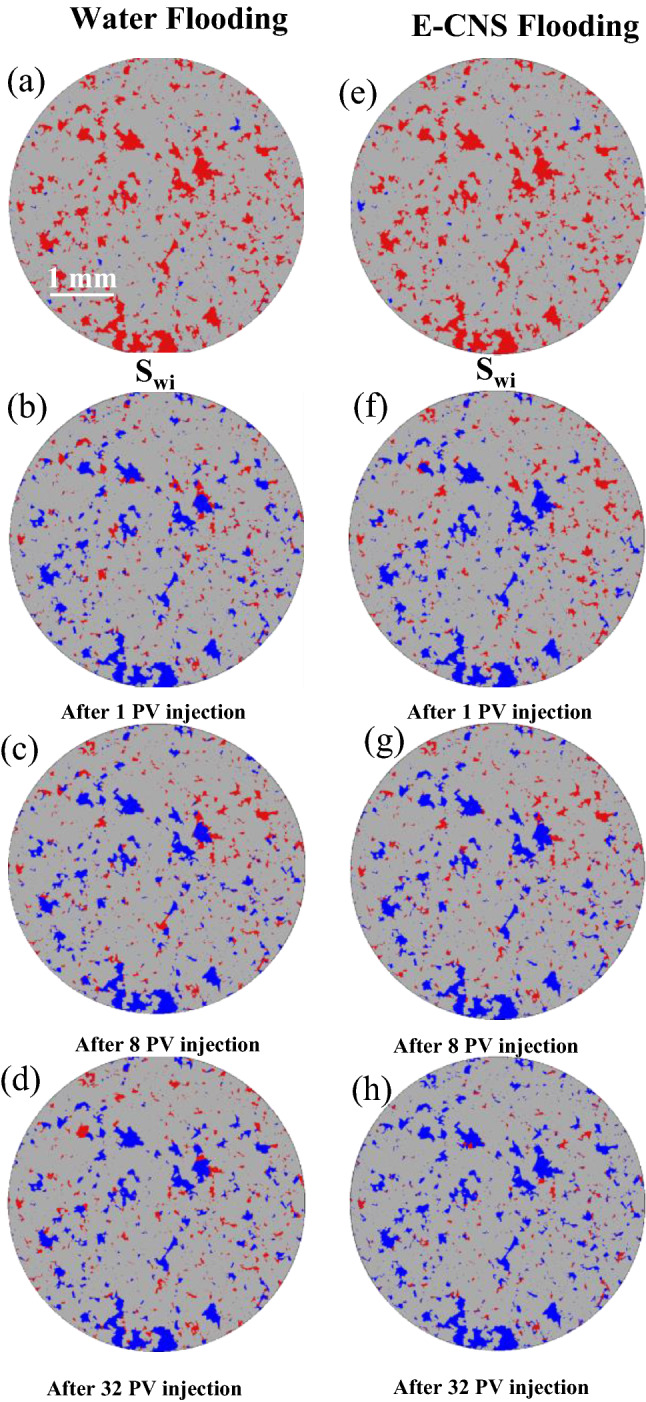
Examples of segmented images at different stages of the core-flooding experiments (blue: brine or nanofluid; red: oil; gray: rock; image resolution: 2.36 microns; image size: 5 mm × 5 mm).

**Figure 6:**
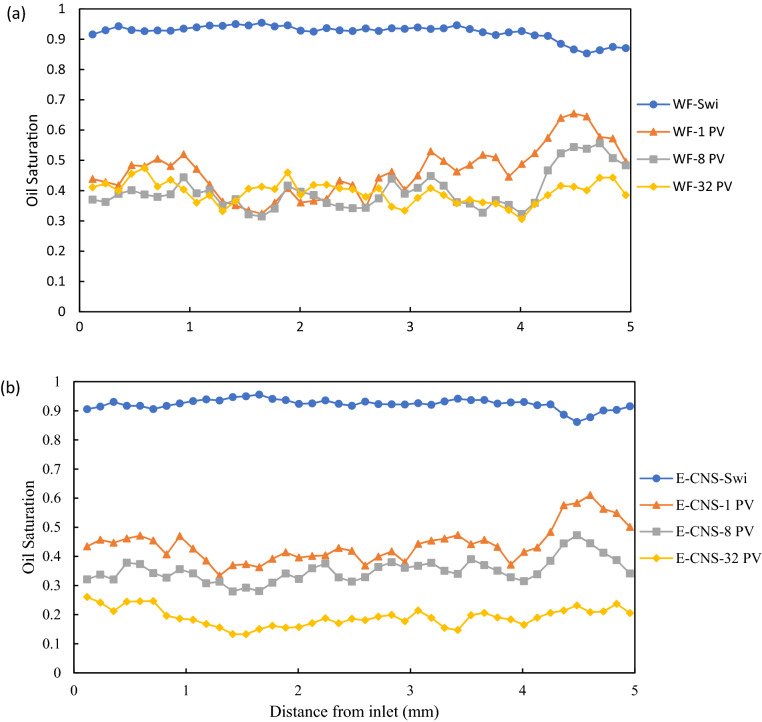
Oil saturation profiles along the length of the core before and after the injection of 1 PV, 8 PV, and 32 PV of displacing fluid during (**a**) water flooding and (**b**) E-CNS flooding.

**Figure 7:**
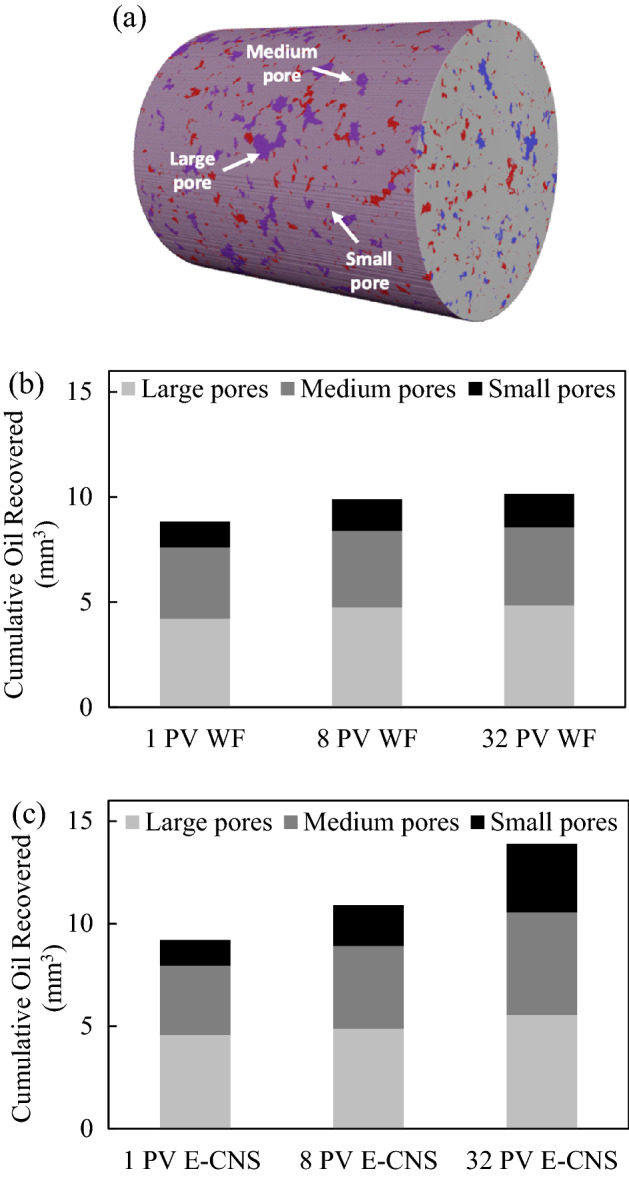
(**a**) Example of 3D segmented rock (4.22 mm in diameter, 5.87 mm in length) showing pores of various sizes. Cumulative oil recovered in large, medium, and small pores at different stages of (**b**) water flooding and (**c**) E-CNS flooding.

Figure 5 shows examples of two-dimensional cross-sectional views of the fluid occupancy configuration in FDL core sample. Figure 5a,e represents the fluid occupancies after the dynamic aging process of the core sample and before the start of the waterflooding and E-CNS flooding tests, respectively, (i.e., steps (iv) and (vi), as shown in the core-flooding procedure in Fig. 2S). The fluid occupancy maps were undistinguishable, confirming that the initial conditions for both flooding sequences were identical. Almost all the large and medium pores (mostly oil-wet) were occupied by oil, while brine only resided in the small pores and crevices (mostly water-wet). The pore-scale fluid occupancy maps confirmed the initial mixed-wet condition of the aged miniature FDL core sample. Figure 6 shows the slice-averaged oil saturation profiles along the scanned length of the core sample at the end of each flow test. The blue curves present the distributions of the initial oil saturation before the waterflooding and E-CNS nanofluids injection were initiated. They show that the initial conditions of these two flow experiments were nearly the same. The initial water saturation (S_wi_) before water flooding was 7.7%, whereas S_wi_ before E-CNS flooding was 7.5%, which translates into 17.63 mm^3^ and 17.60 mm^3^ original oil in place (OOIP), respectively. After the initial water saturation was established, the waterflooding and E-CNS flood tests were initiated to displace the oil phase inside the core sample. The core was subsequently scanned after the injection of 1, 8, and 32 PVs of base brine to investigate the oil recovery process (Fig. 5b–d). The same procedure was repeated during the injection of E-CNS nanofluid (Fig. 5f–h). Figure 7a illustrates an example of a two-dimensional cross-sectional view of the pore map with various pore sizes. As discussed earlier, the pores were categorized by their size to large, medium, and small pores, and since all three groups of pore sizes have similar total volumes, investigating their contributions will help elucidate the EOR displacement mechanisms during the injection of the base brine and E-CNS nanofluid. In the remainder of this section, we discuss the contributions at different PVs of the injected aqueous solutions and the pore-scale mechanisms responsible for the observed recovery trends.

A considerable amount of oil was displaced after the injection of the first PV of the aqueous solution (i.e., the blank brine and E-CNS mixture in the first and second flow tests, respectively). As the displacing fluid was injected under capillary-driven flow, oil inside the pores was gradually displaced in a sequence dictated by the relative magnitude of their threshold entry pressures^48^. Since most of the medium and large pores were oil-wet and small pores were water-wet at the beginning of the tests, the injection of an aqueous solution was a drainage process in the medium and large pores, and hence the oil recovery was dominated by a piston-like pore-scale displacement mechanism. During these pore-scale events, the aqueous phase invaded the center of the pores and left the remaining oil either in the corner of the pores or as layers sandwiched between the aqueous phase in the corners and the aqueous phase in the center (Fig. 5b,f). The displacement, however, in the small pores (mostly water-wet elements) was an imbibition process. As shown in Fig. 6, remaining oil saturations of 45.8% and 43.7% were obtained after waterflooding and E-CNS nanofluid injection, respectively. Since both flow tests were conducted under similar flow rates (i.e., under capillary-dominated flow regime), initial saturation, and wetting conditions, the difference in oil recoveries could be explained by the difference in IFT properties of the aqueous phases. We believe that the lower IFT in the case of nanofluid flooding enabled E-CNS mixture to overcome the entry pressures of more pores, which would also improve its accessibility to even more pore elements. In order to support this statement, we calculated the volumes of oil recovered from pores of different sizes after each flooding and presented the results in Fig. 7b,c. Although some oil was recovered from small pores, most of the amount displaced at this stage was from medium and large pores. Furthermore, E-CNS produced more oil from large pores than the base brine.

**Figure 8:**
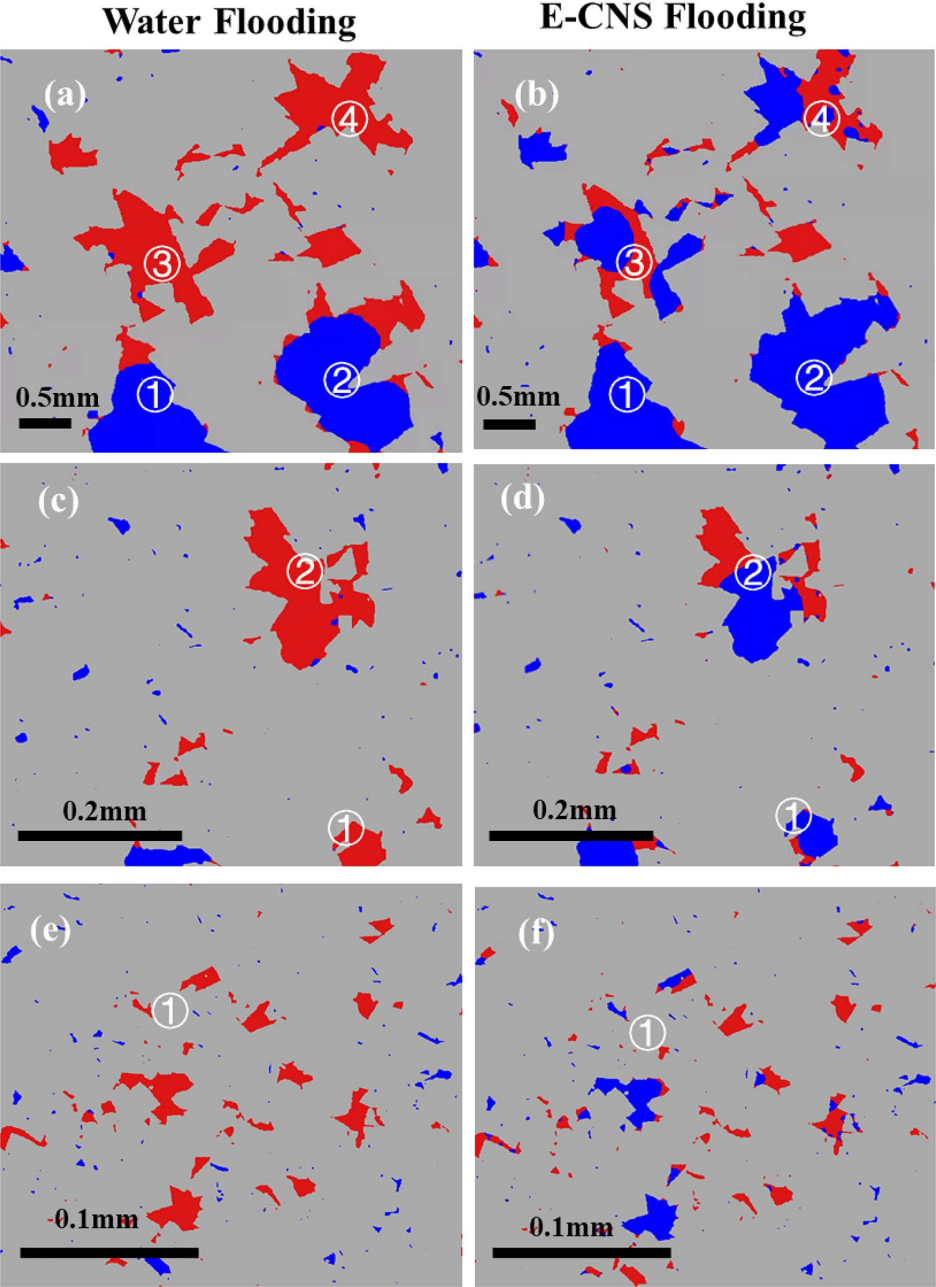
Fluid occupancy maps at different flooding stages. (**a**,**b**) After 1 PV in large pores; (**c**,**d**) After 8 PVs in medium and small pores; and (**e**,**f**) After 32 PVs in small pores.

The fluid occupancy maps illustrating changes in oil saturation and fluid configurations from the initial stages to the end of the flooding process are shown in Fig. 8. Images obtained after injection of 1 PV of each aqueous solution are displayed in Fig. 8a,b. It should be noted that all the marked pores in these two subfigures are large pores. The blank brine invaded only the center of pores 1 and 2, while the E-CNS mixture was able to push the water–oil interface further towards the corner of the pores (i.e., frontal advance displacement). Moreover, the base brine failed to invade pores 3 and 4, whereas E-CNS could invade into the center of these pores. As we will explain in more detail later in this section, these pore-scale events were the reasons why more oil was recovered after E-CNS flooding. Note that pores 1–4 in Fig. 8b were still oil-wet with oil residing in the corners. The higher oil recovery of E-CNS flooding was due to the lower IFT of the oil/nanofluid system (Fig. 2d). Because of the amphiphilic character of the nanosheets, they showed active interfacial properties, which helped the oil/brine interfaces move further in the corners of pores 1 and 2 and invade into the centers of pores 3 and 4. In addition to the above-mentioned events, thinning of the oil layers played a role in the oil recovery at this stage.

Flooding was continued until 8 PVs of the displacing fluid had been injected during each case before a new scan was taken to capture new pore-scale fluid occupancy maps in Fig. 5c,g and to produce the oil saturation profiles. Overall, oil saturation of 40.1% and 34.8% were measured in the core after the blank brine and E-CNS mixture injections, respectively (Fig. 6). The oil saturation after 8 PVs of water injection indicated that only 5.7% more oil was displaced at this stage. This was due to the fact that most of the oil in the large pores had already been displaced after 1 PV of injection, and the threshold water pressures for displacements in oi-wet medium and small pores were higher, which made it difficult for water to invade. However, the oil saturation decreased by 8.9% after E-CNS mixture flooding. As the displacing nanofluid had a lower IFT, it was able to invade the oil-wet medium- and small-sized pores that were previously occupied by oil. These trends can be easily seen when inspecting the fluid occupancy maps of medium pores at this stage, as displayed in Fig. 8c,d. After 8 PVs of water flooding, the water was still not able to invade into medium-sized pore 2, whereas E-CNS nanofluid expelled more than half of the oil residing in this pore. Furthermore, E-CNS displaced oil from the small-sized pore 1, where water flooding showed no impact. The oil saturation and pore occupancy maps verified that the lower IFT was still the dominant factor in enhancing oil recovery. However, the curvatures of the oil–water interfaces and the three-phase contact angles in Fig. 8d were different from those in Fig. 8b, which suggested that the wettability of the pores after 8 PVs of E-CNS flooding had started to change.

After injecting 32 PVs of displacing fluids, a scan was taken in both experiments (Fig. 5d,h), and saturation profiles were plotted in Fig. 6. For the base brine flooding, an overall oil saturation of 45.8% was obtained after 1 PV of water injection, which decreased by 5.7% and 1.3% after the subsequent injections of 8 and 32 PVs, respectively. Figure 7b,c provides the volumes of oil produced from the large, medium, and small-sized pores after 32 PVs of the base brine injection. These data were almost the same as those obtained after 8 PVs of water injection, which implied that increasing the amount of the injected water (at the same flow rate) had an insignificant effect on recovery from the oil-wet core sample. On the other hand, an oil saturation of 43.7% was achieved after the injection of 1 PV of E-CNS nanofluids, which decreased by 8.9% and 15.7% after the subsequent injections of 8 PVs and 32 PVs, respectively (Fig. 6). A substantial amount of oil was displaced at the final stage of nanofluid injection, especially from medium- and small-sized pores (Fig. 7b,c). The fluid occupancy maps in the small pores, provided in Fig. 8e,f, show that the E-CNS nanofluid was able to invade the small pores that were inaccessible by base brine (see pore 1 as an example). The remaining oil saturation was clearly higher after base brine flooding, while E-CNS could invade into more small pores than its water flooding counterparts. Unlike the water floods, the nanofluid was able to alter the wettability of most the pores to a neutral-wet or slightly water-wet state. which will be discussed in detail in the next section.

### Characterization of in-situ wettability

**Figure 9:**
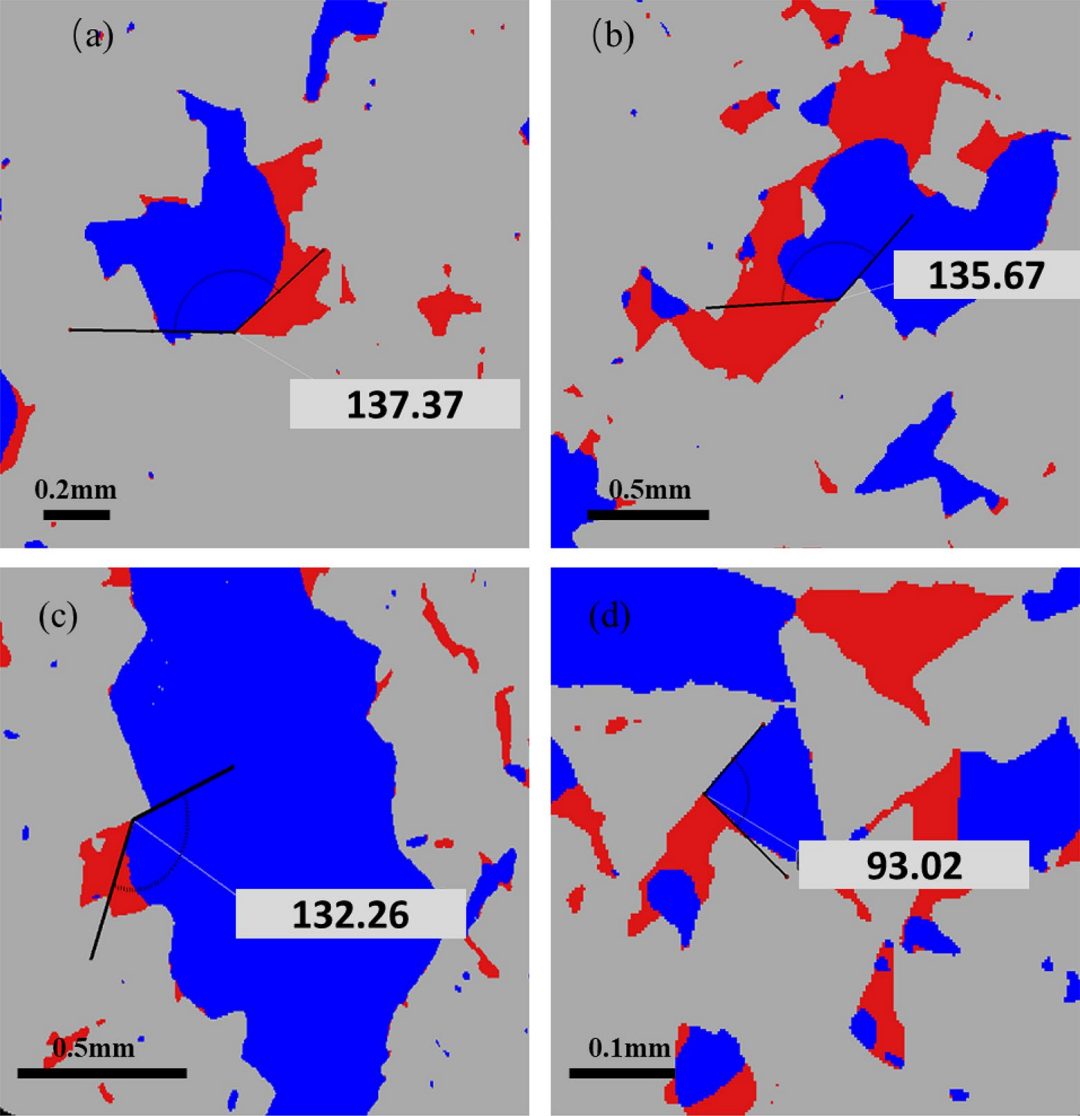
In-situ contact angles at different stages of the experiments. (**a**) Before water flooding, (**b**) before E-CNS flooding, (**c**) after water flooding, and (**d**) after E-CNS flooding.

**Figure 10:**
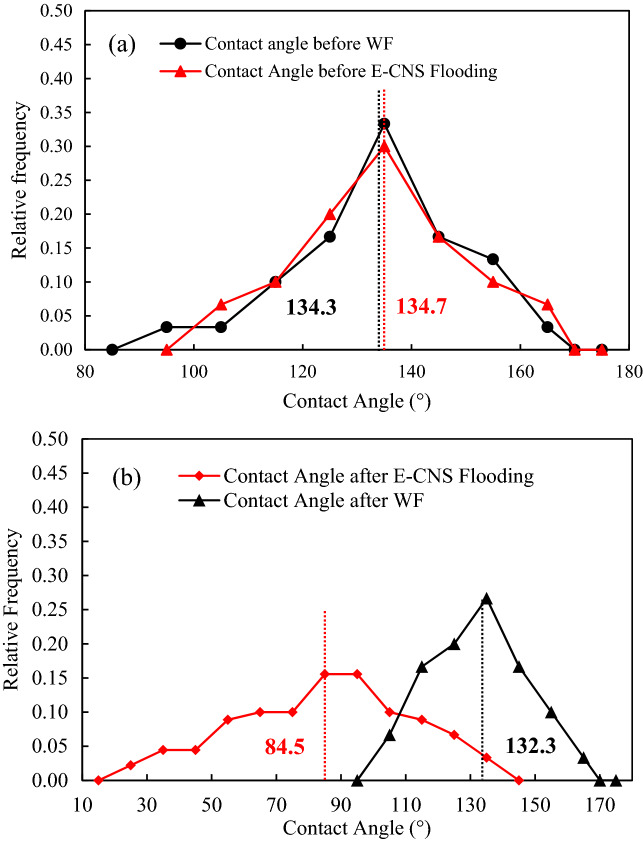
In-situ contact angle distributions (**a**) before the floodings and (**b**) after 32 PV of water and nanofluid injection.

Segmented micro-CT images were utilized to characterize the in-situ wettability reversal at different stages of the experiments. For each pore size (i.e., large, medium, and small), at least 60 in-situ contact angles were measured at randomly selected locations at the end of each flooding step. A detailed description of the measurement procedure can be found elsewhere^49^. Examples of contact angles before and after base brine flooding and nanofluid flooding are illustrated in Fig. 9 and the in-situ contact angle distributions are provided in Fig. 10.

Before the flooding tests, similar average contact angle values were found at the initial water saturation (i.e., 134.3° before waterflooding and 134.7° before nanofluid flooding), which indicated that the initial wetting conditions for both of the flooding experiments were nearly identical (Fig. 10a). At the end of waterflooding, the in-situ contact angles remained almost unchanged with an average value of 132.3°. However, as shown in Fig. 10b, the contact angle distribution after the injection of 32 PV of nanofluid exhibited significant variations, i.e., some pores remained oil-wet while the wettability states of many others were altered to neutral-wet and even water-wet states. Thus, the in-situ contact angles had a broader distribution, where the contact angle values were not symmetrical around the mean value of 84.5°. After nanofluid flooding, the contact angle covered a wide range of values from 25° to 135°, denoting a non-uniform wettability reversal inside the pore space. The wettability reversal is likely due to the E-CNS nanoparticle structuring in the confined wedge film. This behavior results in a structural disjoining pressure gradient at the wedge vertex, which drives the spreading of the nanofluid. The negative zeta potentials of E-CNS (− 3.6 mV) and FDL dolomite (− 27.7 mV) and the relatively low brine salinity suggest minor adsorption of the nanoparticle on the rock, which is in line with adsorption measurements by Quartz Crystal Microbalance with Dissipation monitoring (QCM-D) conducted in our previous work^39^.

**Figure 11:**
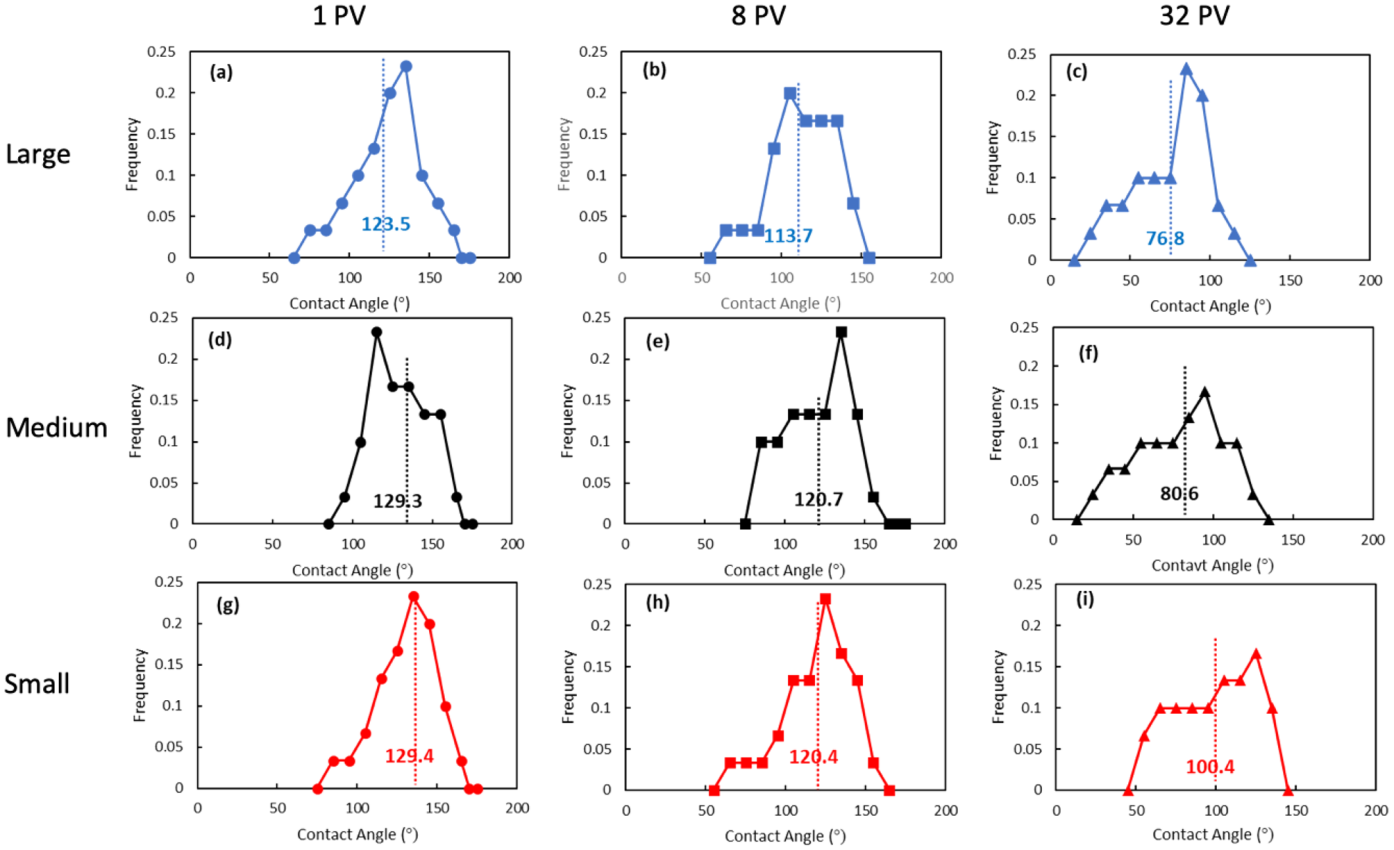
In-situ contact angle distributions in different pore sizes at the end of different E-CNS flooding stages.

To further investigate the degree of wettability alteration by E-CNS in different pore sizes, the in-situ contact angles were measured in the large, medium, and small pores at the different flooding stages. Figure 11a,d,g shows the contact angle frequency distributions for the large, medium, and small pores after 1 PV of E-CNS injection. Changes in the average contact angles in the medium and small pore elements are marginal, whereas the value in large pores decreased from 134.7° to 123.5°. This phenomenon, together with the observations presented in Fig. 7, corroborated the fact that the lower IFT of E-CNS nanofluid was responsible for the higher oil recovery in the early stages of injection. After 8 PV of the nanofluid flooding (Fig. 11b,e,h), the wetting state in all the pores sizes shifted toward reduced oil-wetness. This change was more pronounced in the large pores. At the end of the nanofluid flooding (i.e., after injecting 32 PVs), the average contact angle values decreased tremendously for all pore sizes because of a significant period of fluid-rock contact. In addition, more water-wet pores were observed in large- and medium-sized groups compared with the small one (see Fig. 11c,f,i, which indicated a more efficient wettability reversal. As a result, more oil was recovered between the injection interval of 8 and 32 PVs than that of 1 and 8 PVs (see Fig. 7c and the oil saturation profiles in Fig. 6). Therefore, with sufficient contact, wettability alteration is the dominant mechanism for additional oil recovery during the E-CNS nanofluid flooding. The wettability reversal toward increased water-wetness improved the accessibility of the E-CNS nanofluid to more pore space. Given sufficient time, the wettability of the exposed pore space was reversed toward a more water-wet state, which in turn facilitated the oil recovery from those pore elements.

### Residual oil analysis

**Figure 12:**
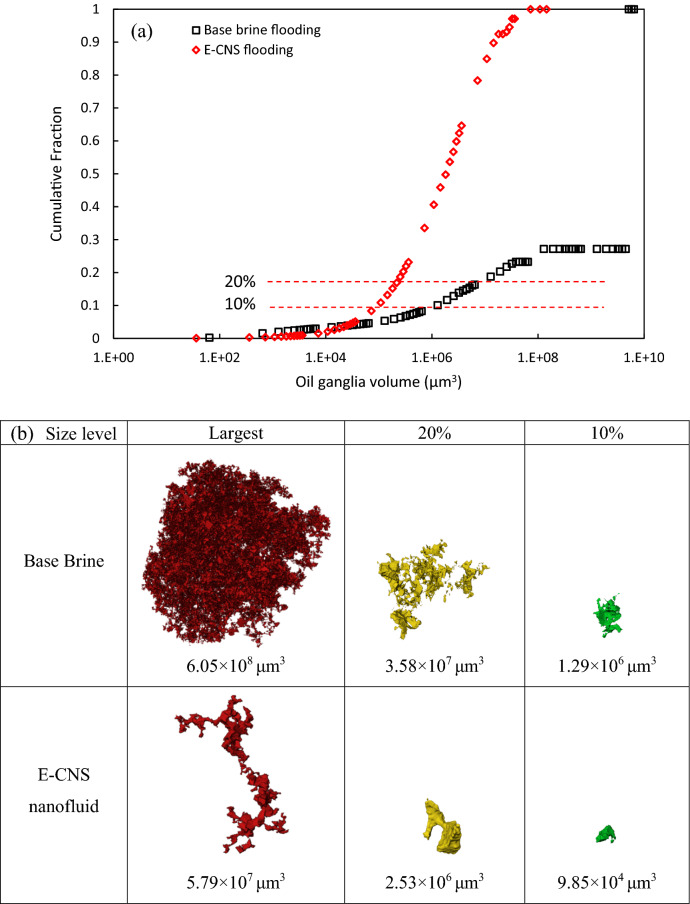
(**a**) Oil cluster size analysis and (**b**) 3D visualization examples of oil cluster.

In the previous section, we found that the E-CNS was able to alter the wettability of oil-wet FDL core to neutral-wet condition, and hence improving the recovery. Considering the pore-scale displacement mechanisms under different wettability conditions, the injection of E-CNS nanofluid may also affect the size distribution of oil clusters after the flooding processes. Therefore, the oil cluster size distribution and an example of three-dimensional (3D) visualization of oil clusters at different size, were analyzed and are shown in Fig. 12. After waterflooding, the largest oil cluster accounted for 73% of the entire oil cluster volume, this behavior was attributed to the oil-wetness of the porous medium. In this scenario, water could only invade into the center of the pores while the wetting oil phase maintained its hydraulic connectivity by the formation of oil layers as well as the presence of oil in some smaller pores. After the injection of E-CNS nanofluid, however, the oil phase lost its connectivity, and as shown in Fig. 12a, the sizes of its cluster reduced significantly (the curve notably shifted to the left). The size reduction is also observed in 3D rendering of the oil clusters in Fig. 12b, in which the largest oil clusters, as well as the oil clusters corresponding to 20% and 10% sizes, are all markedly smaller after nanofluid flooding, in comparison with their counterparts after base brine injection. These results agree with the findings reported by Iglauer et al*.*^50^ and Kuang et al*.*^40^, where larger oil clusters were obtained in oil-wet porous media. Hence, the residual oil cluster analysis also verified the wettability alteration effect of the E-CNS nanofluid.

## Conclusions

The performance of a novel coal-derived E-CNS nanofluid as an EOR agent was investigated at the pore scale by conducting a set of core flooding experiments integrated with x-ray microtomography at elevated temperature and pressure in an oil-wet carbonate. The nanofluid exhibited dual properties of surfactants and nanoparticles by reducing IFT and altering the wettability of the rock. This unique feature enabled it to invade into and alter the wettability of medium and small pores that were otherwise inaccessible to base brine. Dynamic IFT tests showed that the E-CNS reduced IFT between brine and Permian oil by 62.3%, while in-situ contact angle measurements revealed that the E-CNS reversed the wettability of the rock from weakly oil-wet (134.6°) to neutral-wet (85.3°). The combined effect of these two properties was responsible for the higher oil recovery during the nanofluid flooding from the aged carbonate core sample. Furthermore, the in-situ contact angle measurements in large, medium, and small pores at different flooding stages showed that the dominant recovery mechanism was IFT reduction at the early stages (1–8 PV), and wettability alteration at the later stages (8–32 PV) of nanofluid injection. Overall, the engineered carbon nanosheets recovered about 20% more oil than the base brine, demonstrating their potential as effective EOR agents.

## Supplementary information

Supplementary file1.
