# Supplementary material for: Pore-scale experimental investigation of oil recovery enhancement in oil-wet carbonates using carbonaceous nanofluids
Source: Sci Rep. 2020 Oct 16;10:17539. doi: 10.1038/s41598-020-74450-w (PMC7568550; doi:10.1038/s41598-020-74450-w)
Supplement: Supplementary file 1 — Supplementary file1. [file 41598_2020_74450_MOESM1_ESM.docx]

**SUPPLEMENTARY INFORMATION**

**Pore-scale Experimental Investigation of Oil Recovery Enhancement in**

**Oil-wet Carbonates using Carbonaceous Nanofluids**

Bingjun Zhang, Abdelhalim I. A. Mohamed, Lamia Goual,* Mohammad Piri

Department of Petroleum Engineering, University of Wyoming, Laramie, WY 82071, USA

* Email: [lgoual@uwyo.edu](mailto:lgoual@uwyo.edu)), Phone: (307) 766-3278

**Table S1.** Basic petrophysical properties of the cores used in this study.

| No. | L, cm | D, cm | V bulk, cm^3^ | V pore, cm^3^ | Weight, g | Ø, % |  | K, mD |
| --- | --- | --- | --- | --- | --- | --- | --- | --- |
| 1 | 7.236 | 3.794 | 81.806 | 10.58±0.01 | 201.025 | 12.93±0.009 |  | 79.08±0.06 |
| 2 | 7.125 | 3.798 | 80.729 | 10.95±0.03 | 197.262 | 13.56±0.004 |  | 90.62±0.08 |
| 3 | 7.287 | 3.799 | 82.574 | 11.12±0.01 | 201.847 | 13.47±0.02 |  | 80.04±0.08 |
| 4 | 7.048 | 3.796 | 79.771 | 11.18±0.01 | 193.406 | 14.02±0.001 |  | 125.53±0.09 |

**Table S2.** Elemental composition of Fond Du Lac rock by EDX analysis.

| No. | Element | Atom number | Atom% | Abs. error, % |
| --- | --- | --- | --- | --- |
| 1 | Calcium | 20 | 3.08 | 4.43 |
| 2 | Magnesium | 12 | 11.94 | 2.76 |
| 3 | Carbon | 6 | 36.04 | 0.50 |
| 4 | Oxygen | 8 | 47.94 | 0.12 |

**
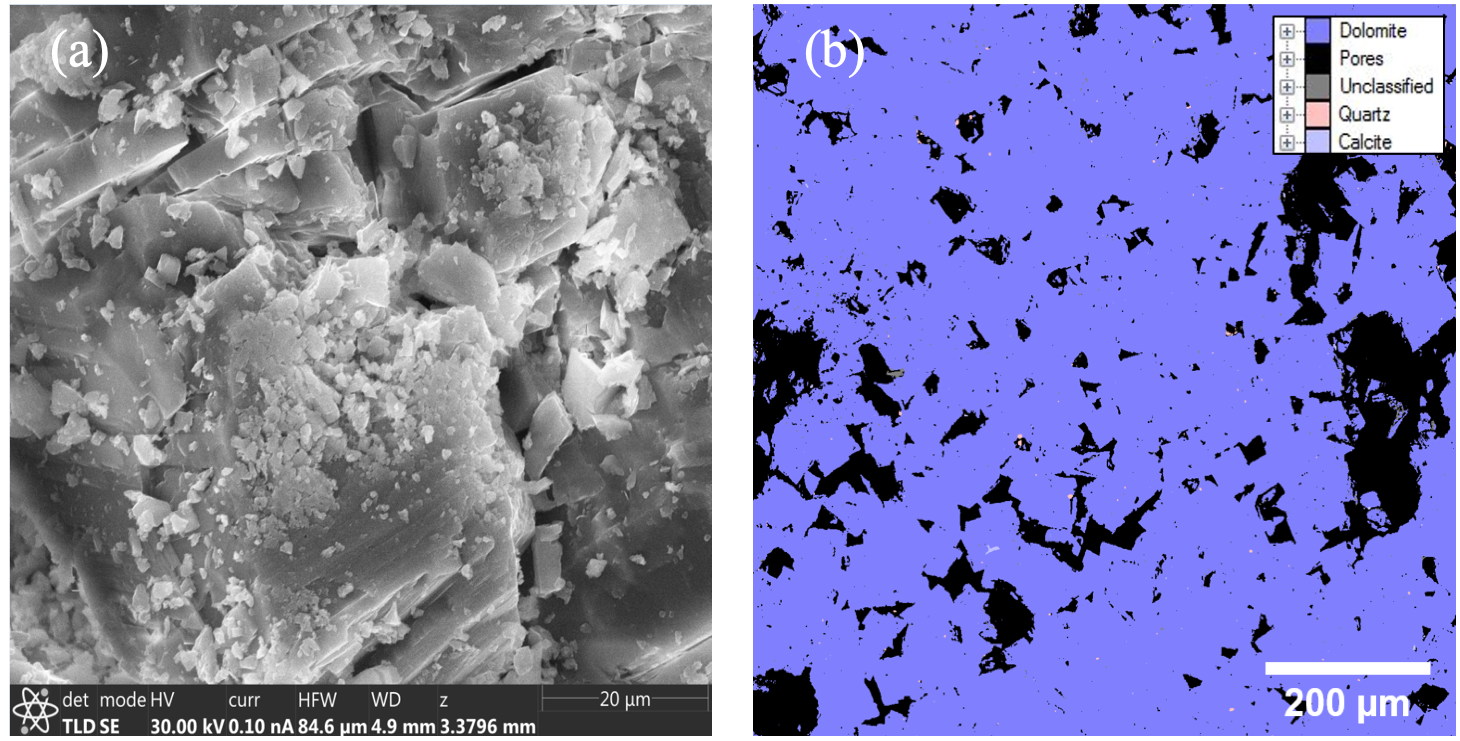
**

**Figure S1.** (a) SEM micrograph and (b) QEMSCAN mineralogy map of Fond Du Lac rock.

**Core-flooding System.** All the micro-scale core-flooding experiments were performed in the miniature Fond Du Lac core sample using HPHT two-phase core-flooding system integrated with a high-resolution X-ray micro-CT scanner (HeliScan^TM^). Schematic diagrams of the experimental setup and procedure are shown in Figure S2. The core-flooding system includes five pulse-free programmable precision dual-cylinder Quizix-5000 pumps, a custom-built high-pressure high-temperature carbon-fiber core holder, six pneumatic valves, and three Rosemount differential pressure transducers with different measuring ranges. Each Quizix-5000 pump has two cylinders that could work both in independent mode and paired mode. The pump has a pressure control accuracy of 0.1 psi at an operating range of 0 to 10,000 psi and has a flow rate control accuracy of 0.0001 cm^3^/min at a working range from -15 to 15 cm^3^/min. In the core-flooding system, three pumps were used as injection pumps for brine, crude oil, and E-CNS nanofluid. These pumps injected the fluids all the way to the bottom of the core holder inside the micro-CT scanner enclosure. Another Quizix pump (i.e., the back-pressure pump) was used to receive the fluid from the top of the core holder. The back-pressure pump was set at the constant pressure receive mode, thereby maintaining a stable pressure inside the core holder. The fifth Quizix pump was utilized to confine stresses on the core sample at a constant pressure by exerting radial stress on the rubber sleeve surrounding the core.

All components outside the micro-CT were connected by HPHT Hastelloy tubing lines, while the components inside the micro-CT were connected by flexible Polyetheretherketone (PEEK) and Polytetrafluoroethylene (PTFE) transparent tubings to reduce potential torque or tension, on the core holder during the tomographic image acquisition, which may affect image quality. Throughout the flow experiments, the temperature of the oven housing the five pumps were kept at 50 °C. All the tubing lines outside the oven and the CT scanner enclosure were wrapped with thermal insulation foams. The injection tubings and core holder inside the micro-CT scanner were also wrapped by heating tapes. The power voltage of the heating tape was carefully adjusted to maintain the temperature inside the core holder at 50 °C.

**Data Acquisition and Image Analysis.** A sub-volume of the core (7.0 mm in diameter and 5.2 mm in length) was scanned at a resolution of 2.36 µm. The location of the scanned area was at 38.6 mm offset from the flow inlet of the core. The scanning position was chosen in the middle part of the 68.4-mm-long core sample to minimize the capillary end effect. Pore-scale fluid occupancy maps were obtained at the end of each experimental step to investigate the underlying mechanism for the superior oil recovery of the E-CNS nanofluid. The scanned images (2200 images per scan) were reconstructed with Q-Mango software and subsequently processed with Avizo 9.4 software. The sample shift during the scan was carefully monitored to guarantee that the same location of the core was scanned each time, thereby ensuring that all comparisons were made for the same pore spaces. In the data analysis procedure, the pore map was first obtained from the dry reference image. The scanned (wet) images with two fluid phases were aligned and registered with the reference image. The re-aligned images were then multiplied with the pore map in order to obtain the fluid occupancy map. Further analyses were conducted based on fluid occupancy at different stages of the experiments. More details on data acquisition and image analysis can be found elsewhere^45^.


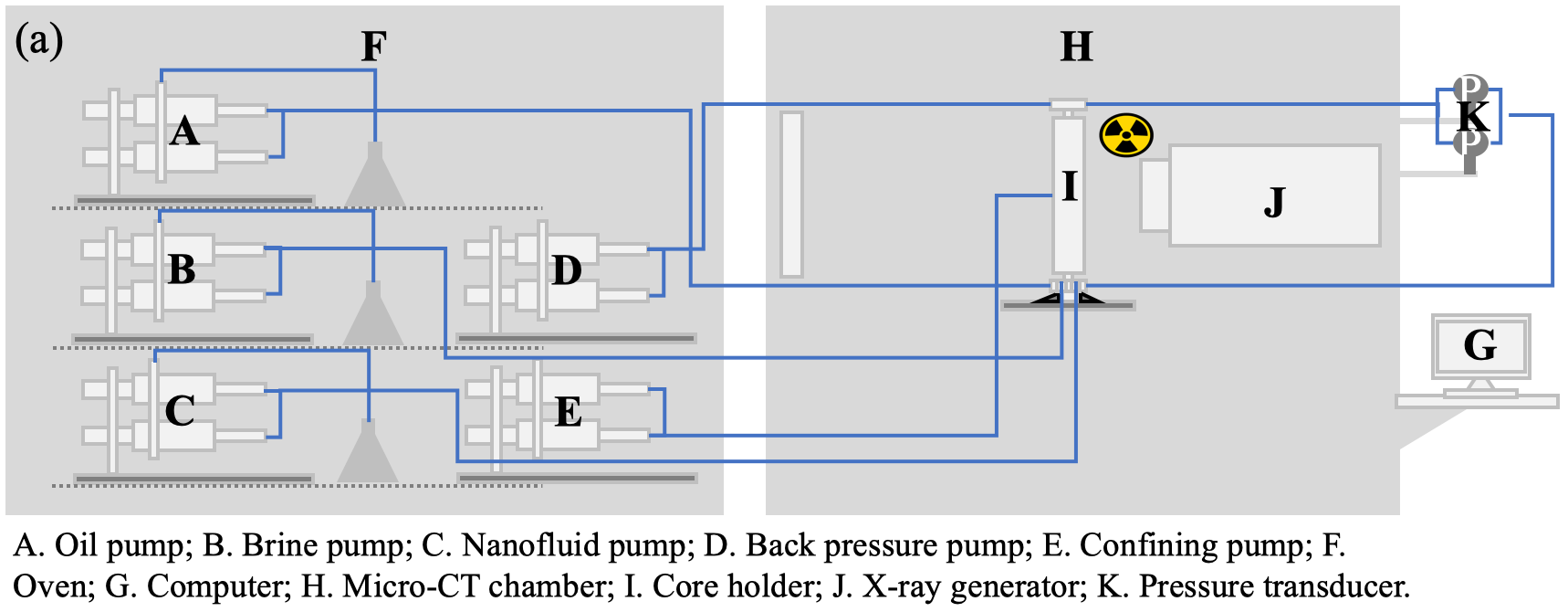


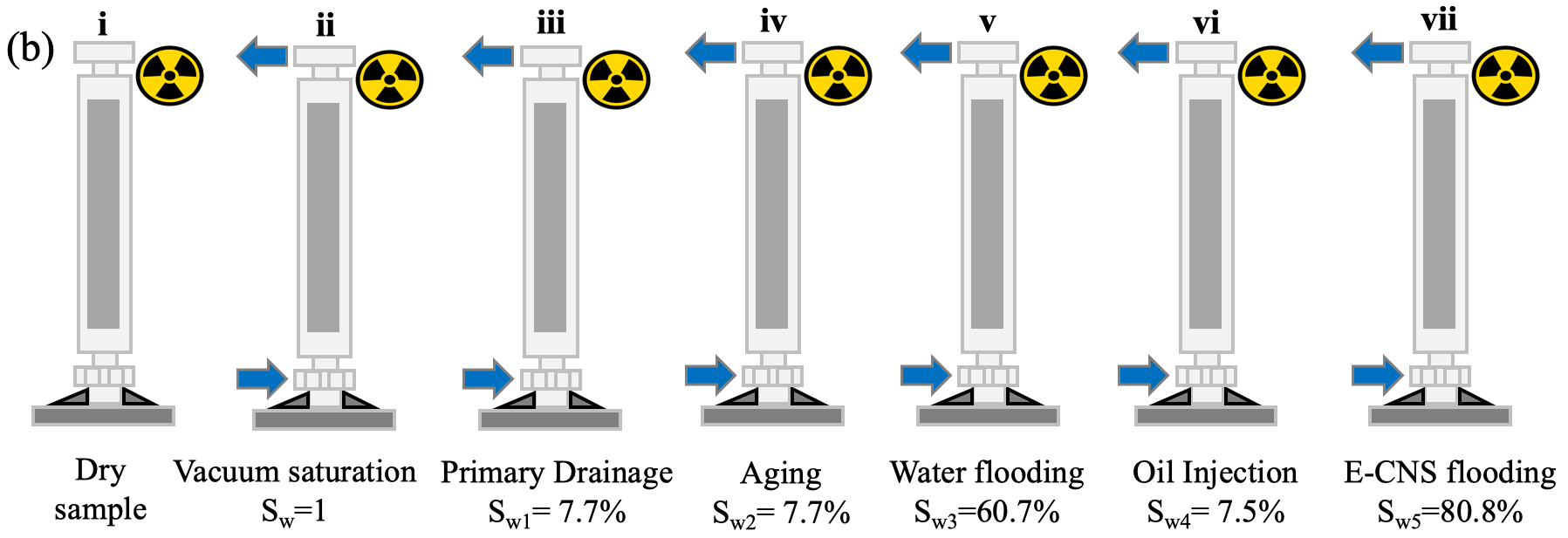


**Figure S2.** Schematic of (a) the experimental setup and (b) flow procedure used in this study.
